# Research landscape and frontiers of non-alcoholic steatohepatitis-associated hepatocellular carcinoma: a bibliometric and visual analysis

**DOI:** 10.3389/fphar.2023.1240649

**Published:** 2023-09-12

**Authors:** Bowen Gao, Zhiheng Chen, Meijie Shi, Yousheng Mo, Huanming Xiao, Yubao Xie, Ming Lin, Xiaoling Chi

**Affiliations:** ^1^ The Second Clinical College of Guangzhou University of Chinese Medicine, Guangzhou, China; ^2^ Department of Hepatology, The Second Affiliated Hospital of Guangzhou University of Chinese Medicine, Guangdong Provincial Hospital of Chinese Medicine, Guangzhou, China

**Keywords:** NASH-associated HCC, non-alcoholic steatohepatitis, hepatocellular carcinoma, tumor-associated macrophages, bibliometric analysis

## Abstract

**Background:** Due to the widespread prevalence of caloric excess and sedentary behavior on a global scale, there is a growing body of epidemiological evidence indicating that non-alcoholic steatohepatitis (NASH) has rapidly become a leading aetiology underlying of hepatocellular carcinoma (HCC). In light of the escalating incidence of NASH-associated HCC (NASH-HCC), it is imperative to mitigate the impending burden. While there has been an increase in global awareness regarding this issue, it has yet to be examined from a bibliometric standpoint. Therefore, this study seeks to provide a comprehensive bibliometric analysis to characterize the evolution of this field.

**Method:** The present study utilized the Web of Science Core Collection (WoSCC) to identify publications pertaining to NASH-HCC over the past 2 decades. Employing Vosviewer 1.6.19, CiteSpace 6.2.R2, and the Analysis Platform of Bibliometrics, the study conducted an analysis of various dimensions including the quantity of publications, countries, institutions, journals, authors, co-references, keywords, and trend topics in this field.

**Results:** A comprehensive analysis of 3,679 publications pertaining to NASH-HCC, published between 1 January 2002 and 1 April 2023, was conducted. The field in question experienced a rapid increase in publications, with the United States serving as the central hub. Collaboration between institutions was more extensive than that between countries. Notably, HEPATOLOGY (*n* = 30,168) emerged as the most impactful journal, and Zobair M. Younossi (*n* = 10,025) as the most frequently cited author in co-citations. The most commonly cited references were KLEINER DE, 2005, HEPATOLOGY (*n* = 630), followed by YOUNOSSI ZM, 2016, HEPATOLOGY (*n* = 493). The author keywords were categorized into three distinct clusters, namely, Cluster 1 (Mechanism), Cluster 2 (Factors), and Cluster 3 (Diagnosis). Analysis of high-frequency co-occurring keywords and topical trends revealed emphasis on molecular mechanisms in current research. “macrophages” and “tumor microenvironment” were active research hotspots at present in this field.

**Conclusion:** A bibliometric analysis was performed for the first time on publications pertaining to non-alcoholic steatohepatitis-hepatocellular carcinoma, uncovering co-research networks, developmental trends, and current research hotspots. The emerging frontiers of this field focused on the macrophages and tumor microenvironment, especially the tumor-associated macrophages, offering a fresh perspective for future research directions.

## Introduction

Currently, primary liver cancer remains an important public health problem all over the world (Llovet et al., 2021). According to the data of the GLOBOCAN (http://globocan.iarc.fr/) estimates, there were 830,180 new deaths in 2020. Hepatocellular carcinoma (HCC), the most common form of primary liver cancer, is the second leading cause of cancer deaths (McGlynn et al., 2021). The escalating prevalence of caloric excess intake and sedentary lifestyle has resulted in non-alcoholic fatty liver disease (NAFLD) emerging as the most rapidly growing cause of HCC in recent times (Younossi et al., 2018; Kulik and El-Serag, 2019). The incidence rate of NAFLD-associated HCC progressively rising in numerous regions globally, particularly in developed countries (Younossi et al., 2018; Huang et al., 2021). Non-alcoholic steatohepatitis (NASH), a progressive form of NAFLD, has been reported to have higher risk for the development of HCC (6, 7). The incidence rate of HCC in NAFLD patients is approximately 0.44 per 1,000 individuals per year, but this rate increases significantly to 5.29 per 1,000 individuals per year during the transition from NAFLD to NASH(8). The transition from NASH to NASH-associated HCC (NASH-HCC) occurs at an annual rate of approximately 2% (Llovet et al., 2023). In the United States, NASH-HCC has become a new growing indication for liver transplantation (Baffy et al., 2012; Wong et al., 2014). It is evident that NASH-HCC will exert a substantial influence on the worldwide social healthcare and economy in the imminent future.

In recent years, increasing studies have been published on NASH-HCC, including epidemic, diagnosis, treatment and mechanism, etc. (Anstee et al., 2019; Dongiovanni et al., 2021; Hindson, 2021) Although a few studies have also analyzed and summarized the research progress of this field, traditional research have not been able to provide a comprehensive overview of global research status and development trends.

Bibliometrics is an emerging area of research in library and information science that employs a range of quantitative and qualitative measures to examine a specific subject matter. Through visual analysis of countries, institutions, journals, and authors, it provides insight into the knowledge structure of a particular field within a global context. Additionally, bibliometric analysis can be used to identify foundational concepts and predict emerging trends by analyzing co-references and co-occurrence keywords, thereby guiding future research directions (Bornmann and Leydesdorff, 2014). In recent years, bibliometric analysis has gained widespread application in the medical field (Shen et al., 2022; Ou et al., 2023; Zhu et al., 2023).

Despite the increasing attention given to NASH-HCC, there has been a lack of relevant bibliometric research conducted on the topic. Therefore, this study seeks to utilize bibliometric analysis and visualization to identify the global cooperation models, research trends, and emerging frontiers of NASH-HCC over the past 2 decades. By comprehending these intricate knowledge relationships, researchers can gain a deeper understanding of the evolving landscape of NASH-HCC research.

## Materials and methods

### Data source and searching strategy

The Web of Science Core Collection (WoSCC) was selected as the data source for retrieval based on its data volume integrity. A search was conducted using the combination of the following keywords and terms: TS = [(“Nonalcoholic steatohepatitis” OR “non-alcoholic steatohepatitis” OR “NASH”) AND (“Hepatocellular Carcinoma” OR “HCC” OR “hepatic carcinoma” OR “hepatoma” OR “hepatocarcinoma” OR “hepatic cancer” OR “liver cancer” OR “liver carcinoma” OR “liver cell carcinoma.”)] The search results were then restricted to the following points: language of English, article types of “articles” or “reviews” and publication date range of 1 January 2002 to 1 April 2023. To reduce the potential bias from frequent database updates, we completed the retrieval process in 1 day on 10 April 2023. Ultimately, a total of 3,679 records were downloaded as “full record and cited reference.” To ensure the accuracy of the bibliometric analysis, two authors (GBW and CZH) independently conducted data extraction using the above search strategy, including titles, abstracts, and publication years, etc. The resulting agreement between the authors was 0.90, indicating a substantial level of concordance (Landis and Koch, 1977). Divergent viewpoints would be discussed and resolved by the team. Before further bibliometric analysis, the thesaurus data were merged.

### Bibliometrics and visualization analysis

**FIGURE 1:**
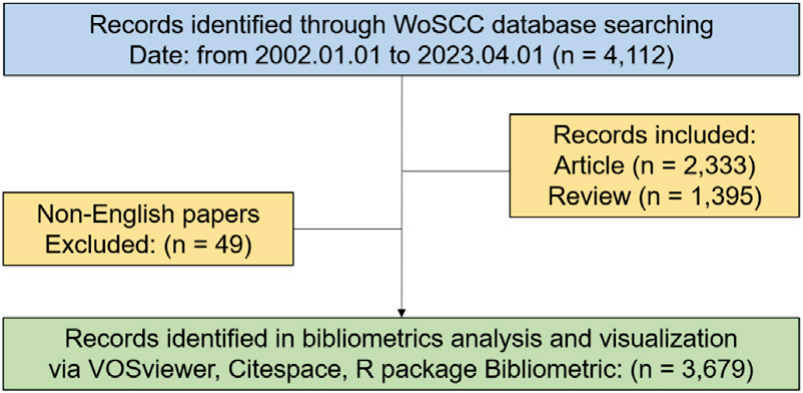
Flowchart of the search process in the study.

We extracted the information from original data, including title, publications, countries, institutions, journals, authors, references and keywords. Data was processed using Microsoft Office Excel 2019 (Microsoft, Redmond, Washington, United States) and a polynomial regression model was constructed to predict the number of publications in 2023. The Analysis Platform of Bibliometrics package was used for descriptive analysis of publications, citations, and growth trends, as well as trend topics. The impact of the study was assessed using a variety of indicators, including publication and citation counts, and the H-index. To visually scrutinize the overall structure of co-research networks, clusters, links between clusters and pathways, and evaluate the influence and significance of publications or key words, VOSviewer 1.6.19 (Leiden University, Leiden, Netherlands), CiteSpace 6.2.R2 (Drexel University, Philadelphia, PA, United States) were utilized (Chen et al., 2006; van Eck and Waltman, 2010; Aria and Cuccurullo, 2017). The detection function in CiteSpace was used to identify the keywords and references with strong citation bursts. Figure 1 showed the flow chart of literature screening and data analysis process.

## Results

### Annual outputs of publications

**FIGURE 2:**
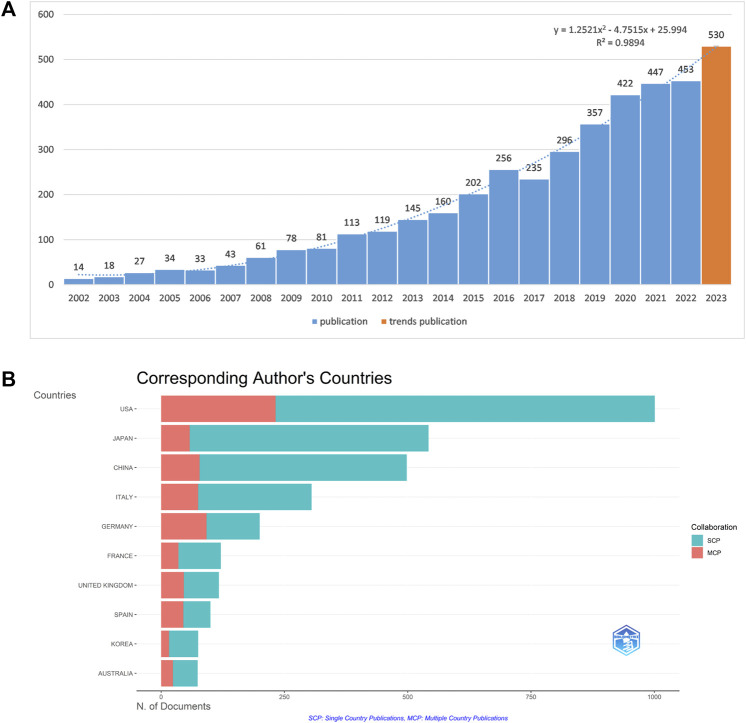
The bibliometric analysis of publication. **(A)** The annual research publication outputs and growth forecast. **(B)** Top 10 countries with most corresponding authors.

Based on the specified search parameters, a comprehensive search of the WoSCC yielded 3,679 publications. A model depicting the growth trend of publications was developed based on historical data (*y* = 1.2521x^2^ - 4.7515x + 25.994, *R* ^2^ = 0.9894, *x*-axis represented the year and *y*-axis the number of publications per year). As shown in Figure 2A, the outputs of publications had a steady increase, with the average growth rate of 9.55%. The annual publication numbers peaked in 2022 (*n* = 453, 12.31%), despite a minor decline in 2017 (*n* = 235, 6.38%). It is estimated that 530 publications would be published in 2023, including 86 publications currently published.

### Distribution of countries and institutions

**FIGURE 3:**
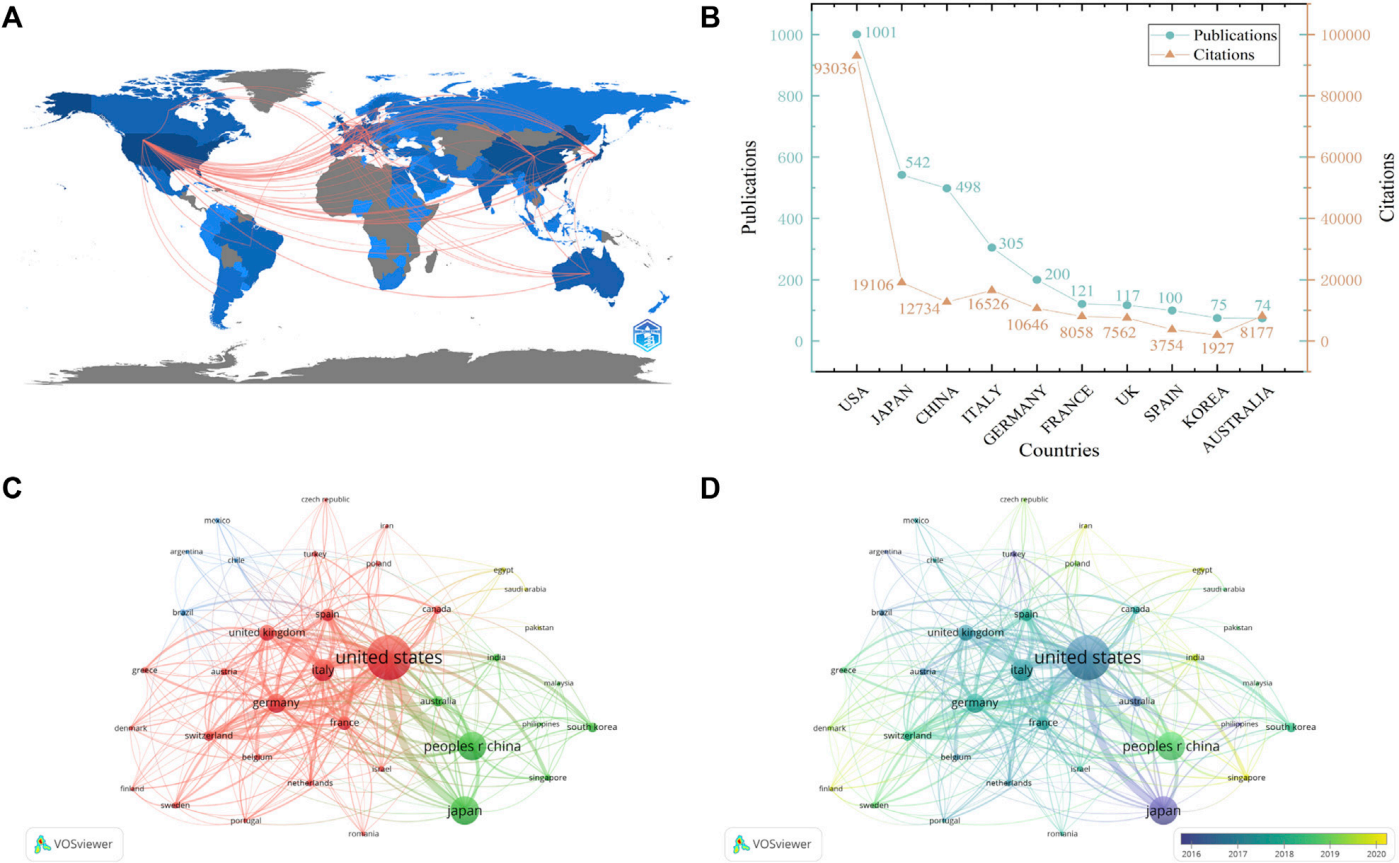
The bibliometric analysis of countries/institutions output. **(A)** The collaboration map among countries. **(B)** The top 10 countries on NASH-HCC research. **(C)** The co-authorship network among countries, with the size and color of circles representing outputs and clusters. **(D)** The overlay visualization of **(A)**, with color representing the average publication year.

In total, these 3,679 publications were produced by 3,785 institutions from 91 different countries. The cooperative relationships among countries were shown in Figure 3A. The top ten countries that contributed to NASH-HCC research was shown in Figure 3B. As for the number of publications, the United States led the pack (*n* = 1,001), followed by Japan (*n* = 520) and China (*n* = 498). The United States ranked first for both single-country publications and multiple-country publications (Figure 2B). Moreover, publications from the United States also received the highest citations (*n* = 93,036). As shown in Figure 3C, co-authorship network among countries was categorized into four clusters: red clusters (the United States and developed European countries), green clusters (Asian countries), blue clusters (South American countries), and yellow clusters (Africa countries). The overlay visualization revealed that China’s publications were concentrated in recent years (Figure 3D). These results revealed that the United States was the central hub of this field, and maintained strong collaborations with China and Japan in Asia, and with Italy, Germany, the United Kingdom in Europe.

**FIGURE 4:**
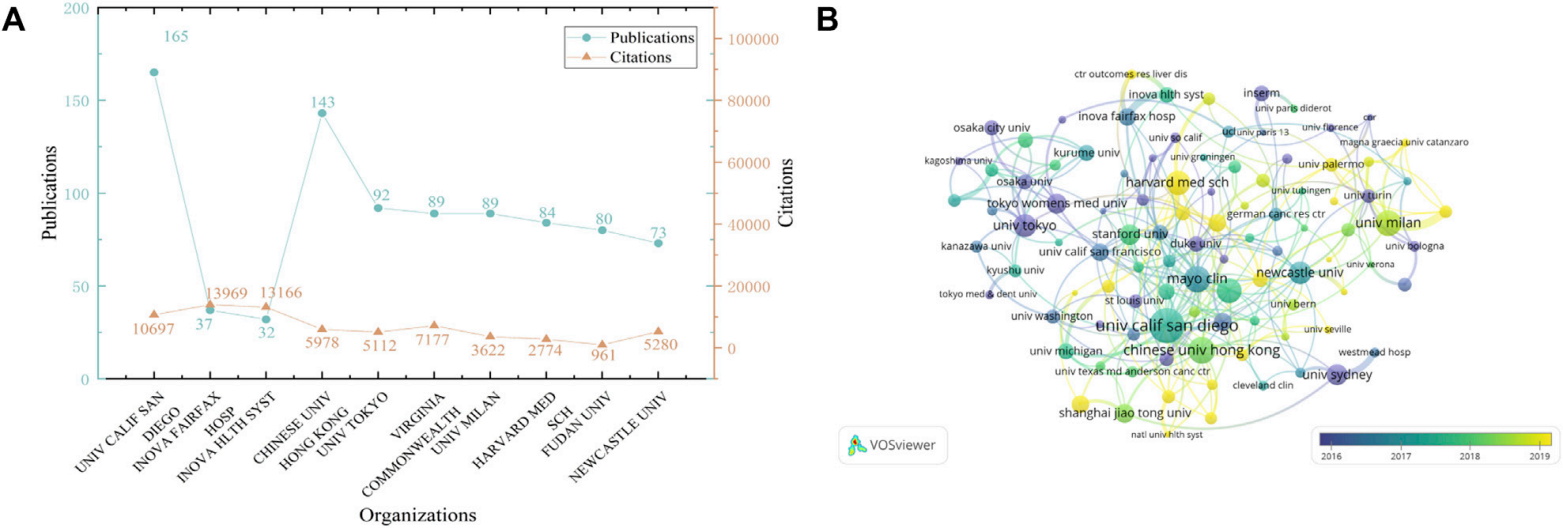
The bibliometric analysis of journals. **(A)** The top 10 institutions on NASH-HCC research. **(B)** The co-authorship network among institutions, with color representing the average publication year.

Furthermore, research progress in this field was being made by an increasing number of institutions, at least 30 publications were produced by the top ten institutions (Figure 4A). The University of California, San Diego (*n* = 165) ranked first in terms of publication outputs, followed by Chinese University of Hong Kong (*n* = 143). Inova Fairfax Hospital (*n* = 13,969) garnered the largest citations. The overlay visualization (Figure 4B) presented highlighted the significant cooperation among institutions and their average publication year. It could be seen that collaboration between institutions was more extensive than that between countries.

### Journals and co-cited journals

**FIGURE 5:**
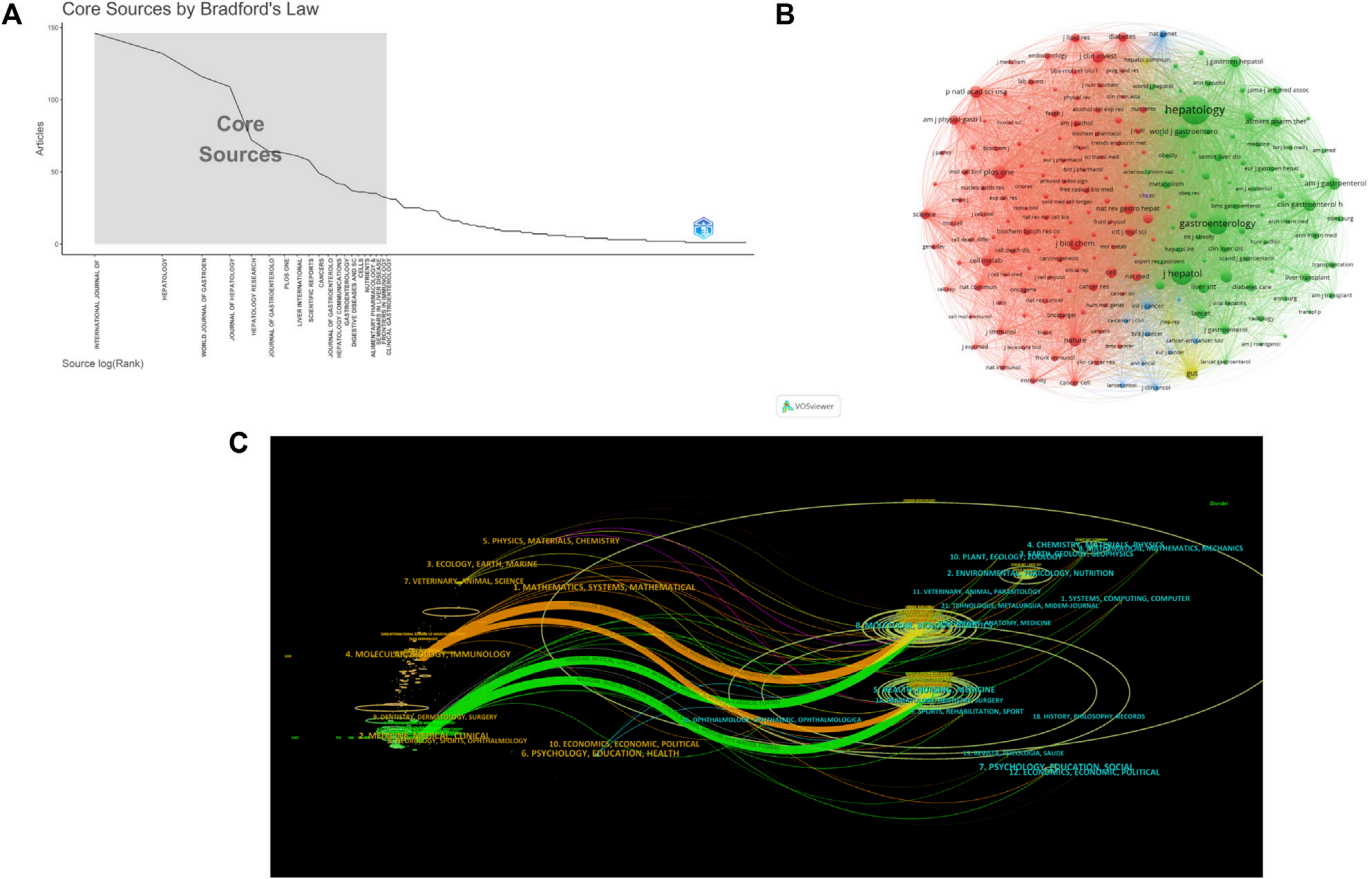
The bibliometric analysis of journals. **(A)** 20 core journals based on Bradford’s Law. **(B)** The co-citation network among journals, with the circle size representing citations. **(C)** The dual-map overlay of journals, with the colored path representing the cited relationship.

These publications were sourced from 793 journals. Twenty journals were identified as core journals using Bradford’s Law (Figure 5A). The INTERNATIONAL JOURNAL OF MOLECULAR SCIENCES had the highest number of publications (*n* = 146), while HEPATOLOGY (*n* = 30,701) was the most cited journal (Table 1). In co-citations network, HEPATOLOGY (publications = 132, citations = 30,701, H-index = 73), JOURNAL OF HEPATOLOGY (publications = 109, citations = 16,345, H-index = 62), and GASTROENTEROLOGY (publications = 41, citations = 12,716, H-index = 36) had significant impacts in this field (Figure 5B). A dual-map overlay of journals highlighted the primary citation connections between citing and cited journals, with the colored path representing the cited relationship. Studies from MOLECULAR, BIOLOGY, GENTICS journals and HEALTH, NURSING, MEDICINE journals were frequently cited by studies from MOLECULAR, BIOLOGY, IMMUNOLOGY journals, as shown by the two orange citation paths. Similarly, the two green paths showed that studies from MEDICINE, MEDICAL, CLINCAL journals were frequently cited by studies from MOLECULAR, BIOLOGY, GENTICS journals and HEALTH, NURSING, MEDICINE journals (Figure 5C). The circle’s size denoted the quantity of publications and authors in a particular field, while the width and height were utilized to determine the author’s ratio.

**TABLE 1:** The top 15 journals on NASH-HCC research.

Journals	Publications	Citations	H index	IF (2021)	JCR Partitions	Country
HEPATOLOGY	132	30,192	73	17.30	Q1	United States of America
J HEPATOL	109	15,437	62	30.083	Q1	NETHERLANDS
GASTROENTEROLOGY	41	13,375	36	33.883	Q1	United States of America
PLOS ONE	63	4,403	29	3.752	Q2	United States of America
GUT	25	4,003	23	31.795	Q1	ENGLAND
WORLD J GASTROENTERO	116	3,985	46	5.374	Q2	United States of America
CLIN GASTROENTEROL H	32	3,580	25	13.576	Q1	United States of America
LIVER INT	61	3,124	25	8.754	Q1	DENMARK
J GASTROEN HEPATOL	64	2,727	30	4.369	Q2	AUSTRALIA
NAT REV GASTRO HEPAT	25	2,619	23	73.082	Q1	United States of America
ALIMENT PHARM THER	35	2,173	23	9.524	Q1	ENGLAND
INT J MOL SCI	146	1790	32	6.208	Q1	SWITZERLAND
J GASTROENTEROL	46	1,658	30	6.772	Q2	JAPAN
HEPATOL RES	72	1,630	27	4.942	Q2	JAPAN
METABOLISM	25	1,434	22	13.934	Q1	United States of America

### Authors and co-cited authors

**FIGURE 6:**
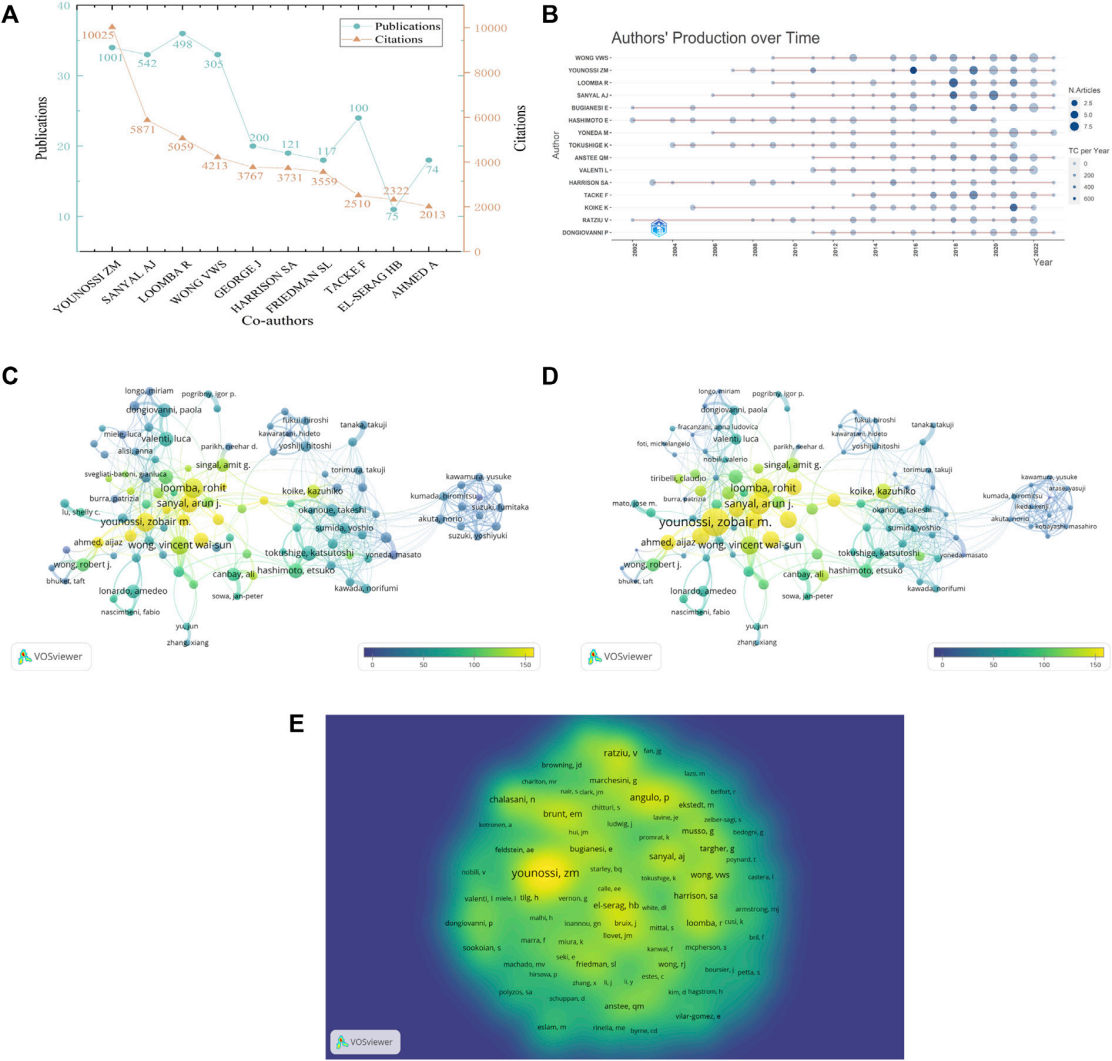
The bibliometric analysis of authors. **(A)** The top 10 authors on NASH-HCC research. **(B)** The annual outputs and citations of authors over time. **(C)** The co-authorship network among authors in terms of outputs, with the size of circles representing outputs and the color indicating the average citations. **(D)** The co-authorship network among authors in terms of citations, with the size of circles representing citations, the color indicating the average citations. **(E)** The co-citation density visualization of authors, the color depth representing citations.

A total of 16,995 authors published relevant papers on NASH-HCC. Four authors published over 30 papers (Figure 6A), namely,: Dr. Rohit Loomba (*n* = 36), Dr. Zobair M. Younosi (*n* = 34), Dr. Arun J. Sanyal (*n* = 33) and Dr. WONG, Wai Sun, Vincent (*n* = 33). It was noteworthy that Dr. Zobair M. Younosi (*n* = 10,025) had the highest total citation count in this field, surpassing Dr. Arun J. Sanyal (*n* = 5,871) and Dr. Rohit Loomba (*n* = 5,059) by a significant margin. Additionally, the annual output and citations of the top fifteen high-yield authors were shown in Figure 6B. The primary co-authorship cluster, situated at the core, consisted of Dr. Zobair M. Younoss, Dr. Rohit Loomba, Dr. Sanyal Arun j, the high number of publications was consistent with the high citations (Figures 6C,D). The size and color depth of the circle were utilized to represent the annual quantity of publications and citations. The density visualization of co-citations analysis was found to be consistent with the above results (Figure 6E).

### Co-citied references

**FIGURE 7:**
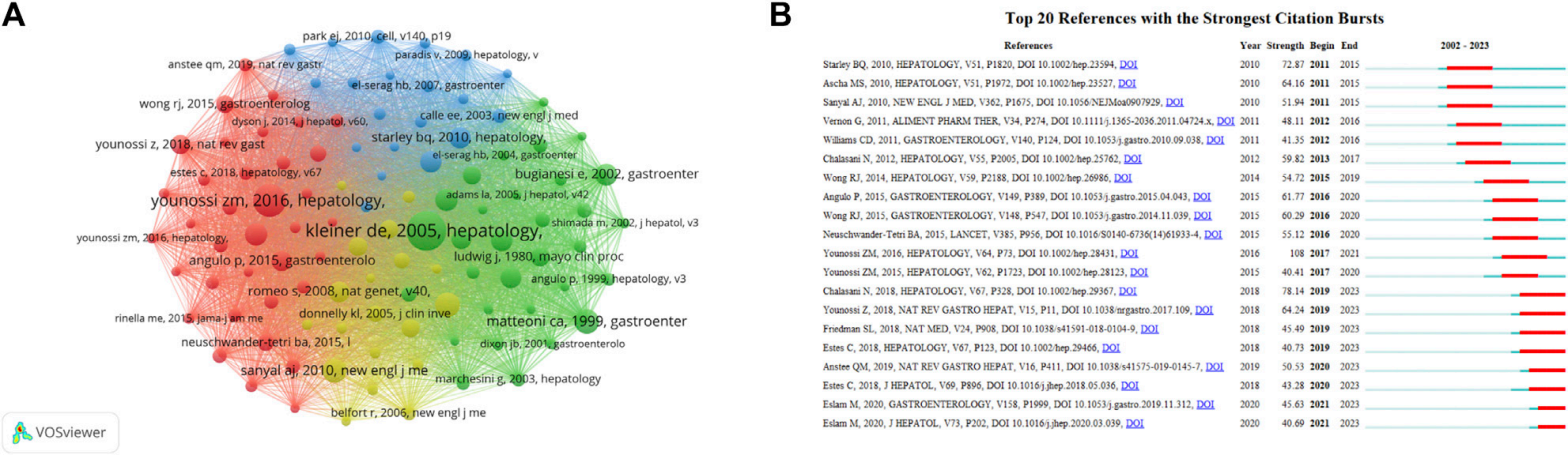
The bibliometric analysis of the references. **(A)** The co-citation network among references, with the circle size representing citations. **(B)** Top 20 references with the strongest citation bursts.

Of the 124,391 co-cited references, 106 were co-cited at least 100 times and visualized mutual cited relationships in Figure 7A. Table 2 showed the top ten co-cited references, the most citations reference was KLEINER DE, 2005, HEPATOLOGY,V41, P1313 (*n* = 630), proposed the NASH histological feature scoring system and the NAS scoring system (Kleiner et al., 2005), followed by YOUNOSSI ZM, 2016, HEPATOLOGY (*n* = 493), V64, P73, estimated the global prevalence, incidence, progression, and outcomes of NAFLD and NASH(8). The top twenty references with the strongest citation bursts were shown in Figure 7B, the bursts detection indicated knowledge structure in research focus within the field. Co-cited references with high citation counts and bursts predominantly centered on guidelines and reviews. The paper entitled CHALASANI N, 2018, HEPATOLOGY, V67, P328, experienced the strongest burst (strength = 78.14) and provided a data-supported approach to the diagnostic, therapeutic, and preventive aspects of NAFLD care (The Diagnosis and Management, 2018).

**TABLE 2:** The top 10 most co-cited references on NASH-HCC research.

Title	Type	Citations	IF (2021)
KLEINER DE, 2005, HEPATOLOGY, V41, P1313, DOI 10.1002/HEP.20701	Clinical Trial	630	17.30
YOUNOSSI ZM, 2016, HEPATOLOGY, V64, P73, DOI 10.1002/HEP.28431	Review	493	17.30
MATTEONI CA, 1999, GASTROENTEROLOGY, V116, P1413, DOI 10.1016/S0016-5085(99)70,506-8	Clinical Trial	348	33.88
DAY CP, 1998, GASTROENTEROLOGY, V114, P842, DOI 10.1016/S0016-5085(98)70,599-2	Editorial	347	33.88
SANYAL AJ, 2010, NEW ENGL J MED, V362, P1675, DOI 10.1056/NEJMOA0907929	Clinical Trial	335	176.08
ADAMS LA, 2005, GASTROENTEROLOGY, V129, P113, DOI 10.1053/J.GASTRO. 2005.04.014	Clinical Trial	311	33.88
CHALASANI N, 2018, HEPATOLOGY, V67, P328, DOI 10.1002/HEP.29367	Review	309	17.30
BUGIANESI E, 2002, GASTROENTEROLOGY, V123, P134, DOI 10.1053/GAST. 2002.34168	Clinical Trial	301	33.88
ANGULO P, 2002, NEW ENGL J MED, V346, P1221, DOI 10.1038/NRDP. 2015.80	Review	294	65.04
ASCHA MS, 2010, HEPATOLOGY, V51, P1972, DOI 10.1002/HEP.23527	Epidemiological Study	292	17.30

### Keywords and trend topics

**FIGURE 8:**
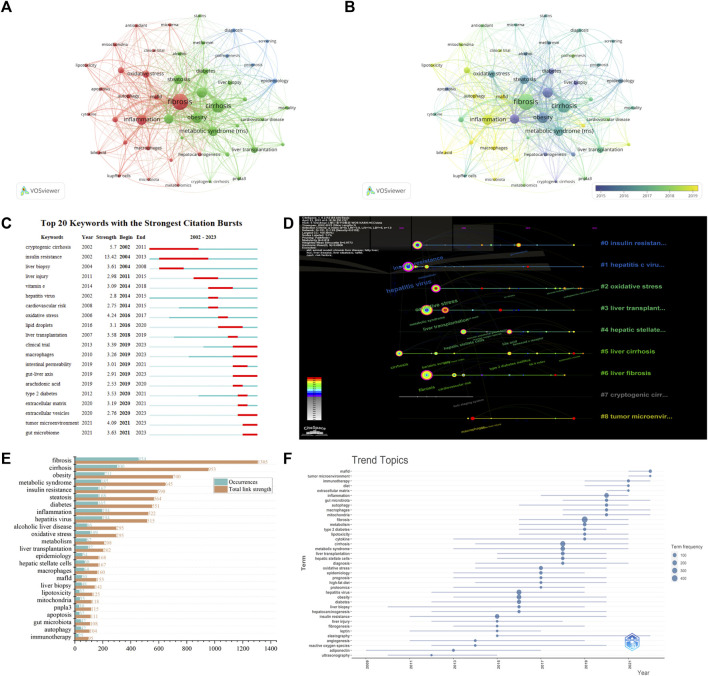
The bibliometric analysis of the keywords and trend topics. **(A)** The co-occurrence network among of author keywords, with the size and color of circle representing amount and clusters. **(B)** The overlay visualization of **(A)**, the color representing the average publication year. **(C)** Top 20 author keywords with the strongest citation bursts. **(D)** The timeline scope of co-citation analysis of author keywords, with each node on the same line representing different years and red nodes representing keywords in **(C)**. **(E)** The top 25 authors keywords in terms of occurrences on NASH-HCC research. **(F)** The trend topics over time.

A cumulative sum of 4,729 author keywords were disseminated. Following the amalgamation of the thesaurus, disease terminologies such as “NASH”, “NAFLD,” and “HCC,” alongside unsuitable keywords such as “liver,” “liver disease,” “risk factors,” “animal model” were expunged. The keywords were partitioned into three distinct clusters in Figure 8A: Red clusters (Mechanism) encompassing “fibrosis” (*n* = 454), “inflammation” (*n* = 194), “insulin resistance” (*n* = 167), “oxidative stress” (*n* = 109), etc. Green clusters (Factors) including “cirrhosis” (*n* = 300), “obesity” (*n* = 211), “metabolic syndrome” (*n* = 185) and “patatin-like phospholipase domain-containing” (PNPLA3, *n* = 30), etc. Blue clusters (Diagnosis) comprising “epidemiology” (*n* = 54), “diagnosis” (*n* = 37), and “screening” (*n* = 25), etc. Important advancements such as fibrosis and cryptogenic cirrhosis in the progression from NASH to HCC continued to be a topic of discussion. “macrophages” (*n* = 64), “metabolic associated fatty liver disease " (MAFLD, *n* = 50), “autophagy” (*n* = 42), etc. were among the high frequency keywords in recent years (Figure 8B). Additionally, the top twenty keywords with the strongest citation bursts were shown in Figure 8C, highlighting “tumor microenvironment” (*n* = 4.09), “gut microbiome” (*n* = 3.63), and “extracellular vesicles” (*n* = 2.79) as current keywords with the strongest citation bursts. Notably, “macrophages” (*n* = 3.26) and “clinical trial” (*n* = 3.39) had remained research hotspots since 2019. Figure 8D depicted the timeline scope and distribution of keywords in this area, revealing trends and interrelationships over time. The nodes on the same line with varying colors represented different years, and the red nodes signified keywords with the strongest citation bursts. Trend topics were consistent with the aforementioned analysis. The top twenty-five author keywords, ranked by occurrence frequency in Figure 8E. Specifically, “tumor microenvironment” and “MAFLD” gained prominence in the past 2 years, while “macrophages,” “immunotherapy,” and “gut-liver axis,” etc. continued to be the subject of intense research interest (Figure 8F). The size of the circles in the figure corresponded to the frequency of occurrence, while the position represented the median frequency. The duration was represented by the line. These findings identified research hotspots and illuminated trends in NASH-HCC. Specifically, it revealed a pronounced emphasis on molecular mechanisms in contemporary research. By merging analysis of keyword bursts and trend topics, the most of active research hotspots were currently identified as “macrophages” and “tumor microenvironment.”

## Discussion

### General information

A thorough exploration of NASH-HCC-related literature was conducted utilizing WOSCC (publications spanning from 1 January 2002 to 1 April 2023). The present bibliometric study encompassed 3,679 papers authored by 16,995 individuals affiliated with 3,785 institutions from 91 countries. These papers were published in 793 journals and feature 4,729 author keywords and 124,391 co-cited references. Notably, the quantity of publications on NASH-HCC is rapidly expanding. It suggests that the understanding of NASH-HCC is gradually improving through continuous in-depth research, making it a vibrant and rapidly developing area of study.

The United States occupied leading position in this field. Notable variations were signified in research levels across different regions. Developed countries seemed more inclined to become research hubs on NASH-HCC, a phenomenon that is somewhat linked to the economic status (Seyda Seydel et al., 2016). The more developed the economy in a region, the more likely it is for people to develop unhealthy habits that lead to NASH-HCC such as caloric excess intake and sedentary behavior. Meanwhile, nations with substantial population sizes, such as China, is encountering significant clinical and research burdens (Estes et al., 2018). As evidenced by the increase in the number of published papers over the last decade, although the total citations of China (*n* = 12,734) remained comparatively low in top ten countries. While it was possible for this situation to improve over time, it is plausible that a dearth of high-quality academic papers may be a contributing factor. Regarding publications and citations, the institutions represented in this field include the University of California San Diego, Inova Fairfax Hospital, University of Tokyo, and The Chinese University of Hong Kong, etc. Evidently, a robust collaborative network existed among research institutions which demonstrated high levels of publications and citations. Nine of the top ten institutions were from the United States, China and Japan, which further confirmed the research centers of field.

The Bradford Law was employed as a common method for assessing core journals. The results revealed that the INTERNATIONAL JOURNAL OF MOLECULAR SCIENCES (publications = 146, citations = 1,790) had the greatest number of publications, yet received a relatively low citation count. Other journals with comparable characteristics were identified, such as CANCERS (publications = 49, citations = 539), CELLS (publications = 36, citations = 484) and HEPATOLOGY COMMUNICATIONS (publications = 42, citations = 880). Contrarily, certain journals with substantial citations, such as NATURE REVIEWS GASTROENTEROLOGY and HEPATOLOGY (publications = 25, citations = 6,002) and GUT (publications = 25, citations = 3,242) were not encompassed within the core journals.

Scholars persisted in their exploration of this field. The elevated publications and citations among authors remained consistent. In 1980, Dr. Ludwig et al. initially identified 20 individuals without alcohol abuse or other liver-damaging factors diagnosed with fatty hepatitis through liver biopsy as NASH [(Ludwig et al., 1980)]. The concept of NAFLD was first introduced and NASH was proposed as a severe subtype by Schaffner and Taler in 1986, as many patients with confirmed fatty liver through liver biopsy did not exhibit liver inflammation (Schaffner and Thaler, 1986). In 2002, Elisabetta Bugianesi et al. discovered that features suggestive of NASH were more prevalent in HCC patients with cryptogenic liver cirrhosis than in age and gender-matched individuals with viral or alcohol-related HCC. This finding had a significant impact on the understanding of NASH-HCC [(Bugianesi et al., 2002)]. As the worldwide prevalence of NASH in the 21st century, cored scholars from the United States led research. Dr. Zobair M. Younossi’s research centered on the epidemic of NASH-HCC, and revealed that NASH was the most rapidly increasing cause of HCC in liver transplant candidates (Younossi et al., 2016; Younossi et al., 2019a; Younossi et al., 2019b). Dr. Rohit Loomba, an esteemed authority in the field of NASH, focused on non-invasive assessment screening from NASH to HCC through the use of advanced imaging modalities, and updated AGA clinical practice on screening and surveillance for HCC in patients with NASH((Loomba et al., 2021; Loomba et al., 2020)). Dr. Sanyal Arun j. developed preclinical models and a diet-induced animal model of NASH-HCC [(Asgharpour et al., 2016; San et al., 2018)]. Dr. Wong Wai Sun, Vincent, has the potential to become a cored scholar in this field as his influence continues to deepen, especially in Asia. It was interesting to note that Japanese scholars led by Etsuko Hashimoto and Norio Akuta established close local co-authorship collaborations, but less cooperation with outsiders.

Our study conducted a bibliometric analysis of NASH-HCC from various perspectives, revealing a pattern of close collaboration among numerous nations, institutions, journals, and authors. The multilateral network of cooperation established through bibliometric analysis and visualization furnishes valuable insights in this field.

### Research hotspots and trend

Bibliometric analysis proved to be a significant tool for evaluating research trends and hotspots from diverse viewpoints. Co-cited references analysis offered the knowledge structure pertaining to NASH-HCC. Our findings revealed that research on risk factors, reviews, and guidelines exhibited the strongest bursts of co-references with the highest citations. However, research on mechanisms had almost never appeared.

The analysis of co-occurring keywords ultimately focused on three areas of research interest, namely, mechanism, factors, and diagnosis, thereby revealing the salient research characteristics of this field. Clinical research has traditionally held a prominent position in the realm of scientific investigation, as evidenced by the prevalence of frequently occurring keywords primarily concentrated within the cluster pertaining to risk factors. The primary pathological mechanism in the disease development of NASH to HCC encompassed “inflammation,” “fibrosis,” etc. (Anstee et al., 2019; Llovet et al., 2023) The frequent occurrence of “insulin resistance” and “oxidative stress” signified the “two hit” pathogenesis theory of NASH, and potentially even the “multiple hit model.” The mechanisms of NASH-HCC were intricately interconnected with the advancement (Buzzett et al., 2016). Simultaneously, risk factors that induced NASH, such as “obesity” and “metabolic syndrome,” as well as those that induced HCC, such as “cirrhosis,” were identified as frequently co-occurring keywords. Researchers investigated the superimposed influence of other risk factors on NASH-HCC or compared the effects of diverse risk factors on HCC [(Marengo et al., 2016; Yip et al., 2022)]. The severity of fibrosis is regarded as the most influential prognostic indicator for establishing a relationship between the advancement of NAFLD/NASH and the occurrence of life-threatening complications. And the incidence of HCC, contingent upon the presence or absence of cirrhosis. However, it should be noted that NASH has the potential to induce liver cancer regardless of the presence or absence of liver fibrosis and cirrhosis (Anstee et al., 2019). In fact, the heightened risk of HCC associated with NASH remains unquantified in several nations. Presently, the diagnosis of NASH necessitates an invasive liver biopsy, which can significantly impede the identification of the increased risk of HCC associated with NASH [(Anstee et al., 2019; Kucukoglu et al., 2021)]. Compared to noninvasive tests for hepatic steatosis and fibrosis, the progress in research on NASH-HCC biomarkers has been comparatively sluggish. This underscores the pressing need for a noninvasive combination of biomarker capable of identifying and assessing potential patients (Wong et al., 2018; Singal et al., 2020). However, the consideration of genetic modifiers is also imperative. Single-nucleotide polymorphisms, such as PNPLA3, have strongly associated with the presence of NAFLD/NASH and the risk of disease progression to HCC. The PNPLA3 polymorphism has been documented to hinder the mobilization of triglycerides from hepatic lipid droplets. Individuals harboring the PNPLA3 polymorphism not only exhibit a heightened susceptibility to NASH but also face a risk of HCC that is more than three times greater (Romeo et al., 2008; Liu et al., 2014).

Visual analysis revealed recent frontiers research predominantly concentrate on basic research. Our findings revealed research frontiers in this field, included “macrophages,” “tumor microenvironment,” “extracellular vesicles,” “gut microbiota,” and “gut-liver axis” etc. It indicated that recent researches concentrated on exploring the pathogenesis of NASH-HCC from diverse perspectives. Additionally, “clinical trial” and “immunotherapy” were identified as the one of recent bursts in keywords and trend topics, respectively. In general, it reflected the research trend in this field: a gradual shift in NASH-HCC research focus from diagnostic methods and examining risk factors to exploring molecular mechanisms and treatment options. However, precise molecular and cellular mechanisms underlying the progression from NASH to HCC have yet to be fully elucidated currently. This process is influenced by a multitude of factors, including the tissue and immune microenvironment, and the microbiome, etc. (Hindson, 2021; Llovet et al., 2023) The exploration of molecular mechanisms was conducted from diverse perspectives and remains an ongoing endeavor, which explained well less mechanism research in the strongest citations bursts and highly co-cited.

Otherwise, “MAFLD” emerged as one of the most frequently co-occurring keywords and latest trend topics in this study. Additionally, it contributed two of the most recent references in the strongest citation bursts: Eslam M, 2020, GASTROENTEROLOGY, V158, P1999 and Eslam M, 2020, J HEPATOL, V73, P202 which proposed a change in the nomenclature from NAFLD to MAFLD [(Eslam et al., 2020a; Eslam et al., 2020b)]. MAFLD is not highlight “steatohepatitis,” and the distinct subtype of NAFLD known as NASH, is eliminated from the terminology. However, the inflammatory activity of NASH is crucial in the development of HCC, and the molecular basis differ significantly with metabolism (Eslam et al., 2020a; Younossi et al., 2021).

### Macrophages and tumor microenvironment

Through an examination of keyword and trend topics, “macrophages” and “tumor microenvironment” were emerged as the most of active research hotspots currently on NASH-HCC. The monocyte-macrophage lineage plays a crucial role in maintaining liver homeostasis and facilitating prompt reactions to hepatic injury. As a fundamental element of liver innate immunity, both tissue-resident (Kupffer cells) and recruited macrophages serve as a significant connection between inflammation and liver cancer (Kazankov et al., 2019; Guilliams and Scott, 2022). It is commonly accepted that macrophages undergo polarization to encompass the classically activated M1 phenotype (pro-inflammatory, anti-tumor), alternative activated M2 phenotype (tumor-promoting effect, immunosuppression), or other activation states. This process is crucial for sustaining homeostasis and reacting to warning signals (Krenkel and Tacke, 2017).

Chronic inflammation has been established as a significant early factor linked to cancer. During the stage of NASH activity, there is an increase in gut-derived endotoxins, lipids and lipid metabolites, as well as the release of pro-inflammatory molecules due to hepatocellular damage and death. This leads to the initiation of an inflammatory reaction by liver-resident Kupffer cells, which recruits blood-derived monocytes. Both of these cell types differentiate into M1 phenotype macrophages, further promoting liver inflammatory response and liver injury. This creates a microenvironment that fosters the generation of genetic mutations. Chronically inflamed liver tissues are prone to malignant transformation and tumor formation in the long run (Kazankov et al., 2019; Pinter et al., 2023).

As tissue cancer occurs, macrophages have a new identity, tumor-associated macrophages (TAMs), which have been utilized as a model to comprehend the correlation between inflammation and cancer (Coussens et al., 2013). The tumor microenvironment (TME) is typified by inflammation and mediators, with TAMs infiltrating the tumor tissue exhibiting high levels of dynamism and heterogeneity within TME. This is primarily evidenced by the capacity of TAMs to acquire distinct phenotypes, metabolic profiles, and functions in response to the influence of the TME. Certain chemotactic factors, such as colony-stimulating factor 1 (CSF-1), activate transcriptional programs that direct TAMs differentiation towards functional skewing of macrophages, resulting in specific phenotypes that are immunosuppressive and tumor-promoting, akin to the “M2-like” phenotype (Noy and Pollard, 2014; Mantovani et al., 2017). However, TAMs may exhibit both M1 and “M2-like” phenotypes, which can coexist within the TME. Certain stimuli can lead to interconversion between these forms of polarization. As such, the function favored by the TAM phenotype is determined by a delicate balance between macrophage activation and suppression, as well as TME [(Tacke and Zimmermann, 2014; Cheng et al., 2022)]. The unbalanced M1/“M2-like” phenotype of macrophages is a critical factor in the development of NASH-HCC.

Furthermore, TAMs have the ability to impede immune pathways and facilitate tumor progression through various mechanisms: TAMs play a significant role in promoting the growth of tumor micro vessels and lymphatic vessels, as well as facilitating tumor cell proliferation. TAMs also can augment the metastasis and infiltration of tumor cells, and contribute to the regulation of immune evasion in tumor cells. Furthermore, TAMs can regulate cell metabolism to support rapid tumor growth (Cheng et al., 2022; Mantovani et al., 2022). Meanwhile, the phenotypic diversity of TAMs results in complex crosstalk with multiple cell types in the liver, including but not limited to liver cancer cells, T cells, endothelial cells, and fibroblasts, ultimately aiding tumor development (Guilliams and Scott, 2022). Notably, extracellular vesicles (EVs) as emerging pathways in complex cellular crosstalk in TME, are also gaining increasing attention (Yang et al., 2022; Chen et al., 2023).

TAMs not only have multiple tumor-promoting functions and abundance, but also are attractive therapeutic targets. Regulatory pathways include inhibiting the generation and promoting exhaustion of TAMs, the inhibition of recruitment, as well as reprogramming through glucose, lipid, and amino acid metabolism from an immune-active to an immune-suppressive state (Mantovani et al., 2017; Xiang et al., 2021; Li et al., 2022). In a personalized medicine approach, TAMs can aid in tailoring the use of cytoreductive therapies and immunotherapy. TAM-focused therapeutic strategies can complement and synergize both chemotherapy and immunotherapy. Overall, the potential regulation of targeting TAMs presents a promising avenue for future research of NASH-HCC in the context. Notably, it is also imperative to acknowledge the significance of considering the immune response of other cell types. For example, tumor-associated neutrophils (TANs) demonstrate comparable characteristics to TAMs that enrich and display functional heterogeneity in NASH-HCC. Efficiently reprogramming of TANs can facilitate their conversion from a tumor-promoting state to an anti-tumor phenotype (Leslie et al., 2022). And there is a correlation between the presence of tumour-infiltrating CD4^+^ regulatory T (Treg) cells and unfavorable outcomes in patients who undergo surgical resection. CD4^+^ T cells exhibit opposing roles in HCC, with both anti-inflammatory and pro-tumorigenic functions reported (Ringelhan et al., 2018). Furthermore, in patients with HCC, the inhibition of natural killer (NK) cell function is mediated by myeloid-derived suppressor cells (MDSCs) and is primarily dependent on NKp30. The compromised function of NK cells in patients with HCC can result in impaired elimination of tumour cells (Hoechst et al., 2009).

Otherwise, “gut microbiota” and “gut-liver axis” displayed in developing a certain level of frequency and discussion enthusiasm in terms of occurrence keywords and trend topics in this field. Recent years have witnessed a burgeoning interest in the study of microbiomes across various bodily systems, with mounting evidence supporting the crucial role of the gut-liver axis in this field pathogenesis. Researchers explored the progression from NASH to HCC development this through the lens of gut microbiota and gut-liver axis, revealing that microbiota-associated molecular patterns may activate macrophages and trigger inflammation, with underlying mechanisms involving alterations in gut microbiota, small intestinal bacterial overgrowth, and changes in intestinal permeability (Frasinariu et al., 2013; Ponziani et al., 2019; Schwabe and Greten, 2020). It is also consistent with the analysis results of our study.

### Strengths and limitations

Our study presented an intuitive, objective, and precise analysis of NASH-HCC publications through the examination of structural relationships, trends, and research hotspots. This is the first study to provide a comprehensive visualization of the data using bibliometric software in multiple dimensions in this field. As such, it has the potential to serve as a valuable resource for scholars and clinicians working in this field. However, the study was inevitably limited by some limitations. Initially, our study might have limitations in terms of its scope, as it solely focused on publications that extracted data from the WoSCC in English, while disregarding other notable search engines such as PubMed, Embase, and Scopus. Consequently, the data might not provide a comprehensive representation of all research conducted in this particular field. Additionally, the possibility of low citation rates might result in the exclusion of some high-quality publications that were recently published. Nevertheless, we intend to address these limitations in future research. Lastly, due to the constraints of visual analysis, we only examined research literature from the past 2 decades in this field, while supplementing past key literature in the discussion. However, it is important to note that the exclusion of premature data did not significantly impact the results.

## Conclusion

Over the past 2 decades, there has been a consistent increase in the number of publications of NASH-HCC. This indicated that research on NASH-HCC is experiencing a vibrant and rapidly evolving phase. The study revealed intricate and multifaceted co-research networks, as well as research trends that have shifted from diagnostic methods and examining risk factors to exploring molecular mechanisms and treatment options. Additionally, this study identified key research hotspots and frontiers, providing valuable insights for researchers seeking to understand the research landscape in NASH-HCC research. “macrophages” and “tumor microenvironment” were currently identified active research hotspots. The investigation into the regulation of TAMs may provide a fresh perspective on potential research avenues for NASH-HCC in the coming years.

## Data Availability

The original contributions presented in the study are included in the article/Supplementary Material, further inquiries can be directed to the corresponding author.
